# Evaluation of Health Equity in COVID-19 Vaccine Distribution Plans in the United States

**DOI:** 10.1001/jamanetworkopen.2021.15653

**Published:** 2021-07-02

**Authors:** Amber Hardeman, Taylor Wong, Joshua L. Denson, Radu Postelnicu, Juan C. Rojas

**Affiliations:** 1 Department of Medicine, Tulane University School of Medicine, New Orleans, Louisiana; 2 Division of Pulmonary, Critical Care, and Sleep Medicine, New York University Grossman School of Medicine, Bellevue Hospital, New York; 3 Section of Pulmonary, Critical Care, and Environmental Medicine, Tulane University School of Medicine, New Orleans, Louisiana; 4 Department of Medicine, University of Chicago, Chicago, Illinois

## Abstract

This cross-sectional study evaluates measures of health equity in COVID-19 vaccination plans in the 50 US states and Washington, DC.

## Introduction

SARS-CoV-2 and the resulting COVID-19 pandemic has affected more than 106 million people worldwide with more than 2.31 million deaths as of February 2021.^1^ Upon the emergency use authorization for a COVID-19 vaccine by the US Food and Drug Administration, the National Academies of Sciences, Engineering, and Medicine developed an overarching framework to assist US policy makers in planning for equitable allocation of COVID-19 vaccines.^2^ Minority populations have approximately 5 times greater risk of adverse COVID-19 consequences related to social determinants of health that may exacerbate patient comorbidities.^4,6^ Equitable distribution would eliminate vaccination disparities while mitigating the disproportionate effect of the COVID-19 pandemic in underserved populations, which are disadvantaged because of limited access to health care, low socioeconomic status, or race. This study aims to determine how every state planned to ensure equitable vaccine distribution.

## Methods

This cross-sectional study was reviewed by the University of Chicago institutional review board and determined to be exempt. Informed consent was waived because no personal or patient data was used. This study followed the Strengthening the Reporting of Observational Studies in Epidemiology (STROBE) reporting guideline.

Investigators analyzed the publicly available COVID-19 vaccination plan for each state and Washington DC, which was accessible on each state’s department of health website. We examined state-specific vaccination distribution models in relation to the US Centers for Disease Control and Prevention COVID-19 Vaccination Program Interim Playbook for Jurisdiction Operations, which was initially created on September 16, 2020.^3^ Each plan was reviewed by 2 authors (A.H. and T.W.) and evaluated with a focus on how all populations would have equitable access to a COVID-19 vaccine.

Data were collected using a standardized questionnaire seeking demographic characteristics and vaccine distribution information for each state. This information included the plan publication date, last updated plan version, vaccination plan committee information, number of distribution phases, demographic characteristics and vaccine allotment quantity per phase, conditions listed as high-risk, use of a health equity task force or diversity strategies, vaccine program monitoring techniques, implementation measures, metrics of distribution success, and communication strategies to maximize distribution (eAppendix I in the Supplement). Data collection and analysis were completed by January 31, 2021.

## Results

In this cross-sectional study, 51 COVID-19 vaccine distribution plans were evaluated, which included 1 plan for each US state and Washington DC. Of the 51 plans, 43 states (84%) created a committee to develop a COVID-19 vaccine distribution plan. A health equity committee was referenced in 20 plans (39%) and not referenced in 31 plans (61%). An implementation committee was referenced in 14 (27%) plans and not referenced in 37 (73%) of the plans. Of the 20 health equity committees, 12 committees reported the types of members involved which included physicians (11 of 12 [92%]), government officials (6 of 12 [50%]), ethicists (4 of 12 [33%]), minority group representations (8 of 12 [67%]), and clergy (5 of 12 [42%]). Of the 51 plans, minority group representatives were present in 8 (16%) of the reported vaccination plans and 26 (51%) of the states collaborating with organizations that serve minority populations. The total number of distribution phases ranged from 3 to 7 with a median of 4. Estimated vaccine allotments per phase and vulnerable patient populations were presented in 14 (27%) and 24 (47%) plans, respectively. High-risk conditions are noted in the Figure. Of the plans, 31 (61%) included partnerships beyond the hospital or medical systems during phase 1, including partnerships with health care and community support services as listed in the Table.

**Figure.**
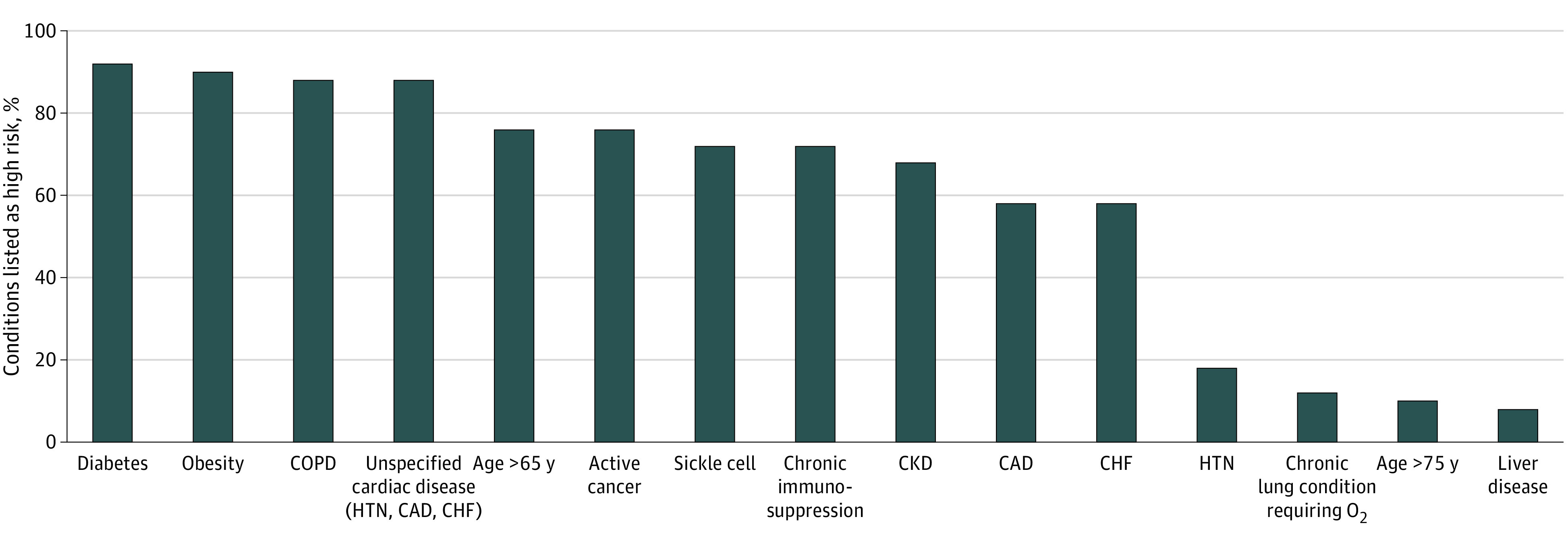
Conditions Listed as High Risk in 50 Vaccination Policies Of 51 vaccination plans, 50 identified high-risk conditions. One state did not specify what conditions they considered to be high risk. CAD indicates coronary artery disease; CHF, congestive heart failure; CKD, chronic kidney disease; COPD, chronic obstructive pulmonary disease; HTN, hypertension.

**Table.** Partnerships Beyond Hospitals or Medical Systems Assisting With Vaccine Plan Implementation

Types of partnerships	Vaccination plans, No. (%)
Health care and community support services (nursing homes, long-term care facilities, clinics, pharmacies)	51 (100)
Homeland and national security	5 (10)
Other critical infrastructure (EMS, communication, technology, banking, finance, shipping)	27 (53)
Correctional facilities	41 (80)
Tribal partners	34 (67)
Educational institutions	28 (55)
Religious or faith-based organizations	27 (53)
Homeless shelters or other service providers	34 (67)
Organizations serving racial and ethnic minority groups	26 (51)
Other	6 (12)

Abbreviation: EMS, emergency medical services.

## Discussion

In this cross-sectional study, most COVID-19 vaccination plans were created without advisement from a health equity committee. This is concerning because there is a disproportionate burden of severe COVID-19 disease and mortality among racial and ethnic minority groups. However, minority group representatives were present in only 67% of health equity committees and 16% of all reported vaccination plans.^4^ States without a health equity committee used partnerships to ensure diversity and equitable vaccine allocation. However, these partnerships also lacked racial/ethnic minority representation, with only 51% of states collaborating with organizations serving minority populations. Additionally, ethicists can identify priority populations during the national allocation of limited COVID-19 vaccines; however, health equity committees were not likely to include an ethicist.^5^ A limitation of this study is that 41 vaccination plans were not finalized at the time of review and may have been modified after initial distribution phases were completed. Future studies should evaluate whether current protocols may lead to inequities in vaccine distribution during subsequent phases and how inequities will be addressed in future vaccination plan updates.

## Notes

Supplement. **eAppendix.** Standardized Questionnaire to Evaluate for Each State Plan Used by Reviewers
