# Supplementary material for: Evaluation of Health Equity in COVID-19 Vaccine Distribution Plans in the United States
Source: JAMA Netw Open. 2021 Jul 2;4(7):e2115653. doi: 10.1001/jamanetworkopen.2021.15653 (PMC8254130; doi:10.1001/jamanetworkopen.2021.15653)
Supplement: Supplement. — eAppendix. Standardized Questionnaire to Evaluate for Each State Plan Used by Reviewers [file jamanetwopen-e2115653-s001.pdf]

Supplemental Online Content

Hardeman A, Wong T, Denson JL, Postelnicu R, Rojas JC. Evaluation of health equity in COVID-19 vaccine distribution plans in the United States. *JAMA Netw Open*. 2021;4(7):e2115653. doi:10.1001/jamanetworkopen.2021.15653

eAppendix. Standardized Questionnaire to Evaluate for Each State Plan Used by Reviewers

© 2021 Hardeman A et al. *JAMA Network Open.*

COVID-19 Vaccine Allocation

COVID-19 Vaccination Policy State Survey

* 1. Which State?

2. Initial Date on State Allocation Policy

Date / Time

Date

MM/DD/YYYY

3. Final Revision Date on Allocation Policy?

Date / Time

Date

MM/DD/YYYY

4. Vaccination plan developed by committee/task force?

- Yes
- No

5. **If no committee, who was responsible for vaccination plan?**

6. Health equity task force used by state?

- Yes
- No

© 2021 Hardeman A et al. *JAMA Network Open.*

7. Health equity committee members if known

- ☐ Physician
- ☐ Underrepresented Patient Representative
- ☐ Government Official
- ☐ Clergy Member
- ☐ Ethicist

8. Number of Phases Listed in States Plan

- ☐ Phase 1
- ☐ Phase 5
- ☐ Phase 2
- ☐ Phase 6
- ☐ Phase 3
- ☐ Phase 7
- ☐ Phase 4

9. Estimated number of vaccine allotments were provided for each phase?

- ○ Yes
- ○ No

10. Estimated numbers of vaccine allotment were provided for each prespecified “at-risk” group?

- ○ Yes
- ○ No

© 2021 Hardeman A et al. *JAMA Network Open.*

11. Phase 1 (only include the first “phase” i.e. phase 1a, not 1b) included which of the following (check all that apply):

- ☐ Hospital personnel with direct covid exposure (Hosp_Direct)
- ☐ Hospital personnel with no direct covid exposure (Hosp_Indirect)
- ☐ Nursing home personnel with direct covid exposure
- ☐ Nursing home personnel with no direct covid exposure
- ☐ Nursing home residents
- ☐ Hospitalized patients without COVID diagnosis
- ☐ EMS
- ☐ Firefighters
- ☐ Police
- ☐ Homeless
- ☐ Urgent Care Healthcare Workers
- ☐ Other outpatient care centers (psych, surgical centers, dentists, optometrist, etc)
- ☐ Jail or Corrections officers
- ☐ Jail or Corrections inmates
- ☐ Pharmacists with direct patient care exposure
- ☐ Pharmacists with no direct patient care exposure
- ☐ Essential workers who cannot work remotely (i.e. food processing/grocery workers, water/energy/waste sector workers, etc.)
- ☐ General public with high risk conditions
- ☐ General public with age>65
- ☐ General public with age>75
- ☐ Caregivers of at-risk individuals
- ☐ Teachers/School staff
- ☐ Transportation (i.e. bus drivers, transit operators, etc)
- ☐ Laboratory personnel who have exposure to specimens from COVID-19+ patients
- ☐ Morgue/mortuary workers in contact with COVID-19+ bodies
- ☐ People living on college/university campuses
- ☐ People living in congregate living facilities or group homes
- ☐ People who are under/un-insured
- ☐ Employees caring for adults or children (i.e. home aides, child care providers)

12. Which conditions represent "high-risk" group in the state's vaccination policy?

- ☐ Diabetes
- ☐ Obesity
- ☐ HTN
- ☐ HLD
- ☐ Active Cancer
- ☐ COPD
- ☐ CKD
- ☐ Chronic Lung Conditions requiring Oxygen
- ☐ Chronic Immunosuppressed
- ☐ CHF
- ☐ CAD
- ☐ Sickle Cell
- ☐ Liver disease
- ☐ Age>=65
- ☐ Unspecified cardiovascular disease (HTN, CAD, CHF
- ☐ Age >=75

13. How was diversity included in vaccination plan if It was not published in guidelines?

- ☐ Gender
- ☐ Age
- ☐ Ethnicity
- ☐ Living Situation
- ☐ Race
- ☐ Socioeconomic status

14. Was critical population mapping used to evaluate areas of highest need for vaccination?

- ◯ Yes
- ◯ No

15. 14. Were metrics of success and/or distribution fidelity proposed (Vaccine program monitoring)?

- ◯ Yes
- ◯ No

16. Was second dose reminder system referenced in state policy?

- ◯ Yes
- ◯ No

17. Was second dose appointment made at time of intial vaccination per state policy?

- ◯ Yes
- ◯ No

18. What measures or technology were being used to ensure patient's return for second vaccination?

- ☐ Vaccination record card
- ☐ Alerts from the state’s Immunization Information System (IIS)
- ☐ Other postcard/mailing system (excluding IIS)
- ☐ Other texting/phone messaging Platform (excluding IIS)
- ☐ Other email Services (excluding IIS)
- ☐ Other phone calling reminder service (excluding IIS)

19. Were implementation measures proposed?

- ◯ Yes
- ◯ No

20. Was an implementation committee established?

- ◯ Yes
- ◯ No

21. Were partnerships and/or vaccine promoters identified to maximize distribution to the community?

- ☐ Health care and community support services (i.e. nursing homes, long term care facilities, clinics, pharmacies)
- ☐ Homeland and national security
- ☐ Other critical infrastructure (i.e. EMS, communication technology, banking and finance, shipping, etc.)
- ☐ Correctional Facilities
- ☐ Tribal partners
- ☐ Educational Institutions
- ☐ Religious or faith-based institutions
- ☐ Homeless shelters/homeless service providers
- ☐ Organizations serving racial and ethnic minority groups
- ☐ Other

22. During phase 1, were there partnerships outside of hospital/medical systems? (i.e. medical societies to reach eligible candidates who don't have a direct link to a hospital system?)

- ◯ Yes
- ◯ No

23. Emergency vaccine storage plan? (i.e. power outages, severe weather, after hours access)

- ◯ Yes
- ◯ No

24. Does the state have/set up specific vaccination program training requirements to ensure efficiency in vaccine operations? (i.e. orientation, position and equipment specific training)

- ◯ Yes
- ◯ No

25. Is a COVID-19 death summary listed for the state?

- ◯ Yes
- ◯ No

26. Are there specific communication strategies/guidelines for the COVID-19 effort listed?

- ◯ Yes
- ◯ No

27. If communication strategies are noted in state plans, what are they?

- ☐ Press releases, scheduled conferences/calls, etc.
- ☐ Electronic messaging boards or alerts
- ☐ Social media platforms or other traditional media campaigns (i.e. digital, print, broadcast, etc.)
- ☐ Other
